# Carbon Quantum Dots Interactions with Pyrogallol, Benzoic Acid, and Gallic Acid: A Study on Their Non-Covalent Nature

**DOI:** 10.3390/nano15181457

**Published:** 2025-09-22

**Authors:** Laura Andria, Giancarlo Capitani, Barbara La Ferla, Heiko Lange, Melissa Saibene, Luca Zoia, Barbara Vercelli

**Affiliations:** 1 Istituto di Chimica della Materia Condensata e di Tecnologie per l’Energia, CNR-ICMATE, Via Cozzi, 53, I-20125 Milano, Italy; 2 Dipartimento di Scienze dell’Ambiente e della Terra, Università degli Studi di Milano-Bicocca, Piazza della Scienza, 1, I-20126 Milano, Italy; giancarlo.capitani@unimib.it (G.C.); barbara.laferla@unimib.it (B.L.F.); heiko.lange@unimib.it (H.L.); luca.zoia@unimib.it (L.Z.); 3 Biochemical Process Engineering, Division of Chemical Engineering, Department of Civil, Environmental and Natural Resources Engineering, Luleå University Technology, SE-97187 Luleå, Sweden; 4 NBFC-National Biodiversity Future Center, I-90133 Palermo, Italy; 5 Piattaforma di Microscopia, Università degli Studi di Milano-Bicocca, Piazza della Scienza 3, I-20126 Milano, Italy; melissa.saibene@unimib.it

**Keywords:** carbon quantum dots, food preservatives, benzoic acid, gallic acid, pyrogallol, electronic interactions of aromatic systems

## Abstract

Understanding the interactions between carbon quantum dots (**CDs**) and promising food preservatives (**FPs**), like pyrogallol (**PG**), benzoic acid (**BA**), and gallic acid (**GA**), is highly relevant. This knowledge is crucial for designing **CD**-based sensors capable of determining the safe levels of these molecules in food and beverages. Additionally, such sensors could be exploited in the development of sustainable, intelligent packaging that controls food shelf life. Based on those considerations, in this study, we post-functionalized blue-emitting **CDs**, prepared according to a synthetic approach previously developed, with the **FP** molecules **PG**, **BA**, and **GA** to obtain **CD-(FP)** systems. UV-vis absorption and FTIR spectroscopy confirmed the presence of the **FP** molecules on the **CD** surface. The appearance of a new vibrational band at 1196 cm^−1^ in the FTIR spectra of all **CD-(FP)** systems suggested that the three **FP** molecules interact with the **CD** surface via electronic interactions between the aromatic and delocalized electron systems. Further electrochemical analyses of the **CD-(PG)** and **CD-(GA)** systems show that the interactions between **PG** and **GA** benzene rings and **CDs** prevent their oxidation to the corresponding quinone forms.

## 1. Introduction

**Chart 1:**
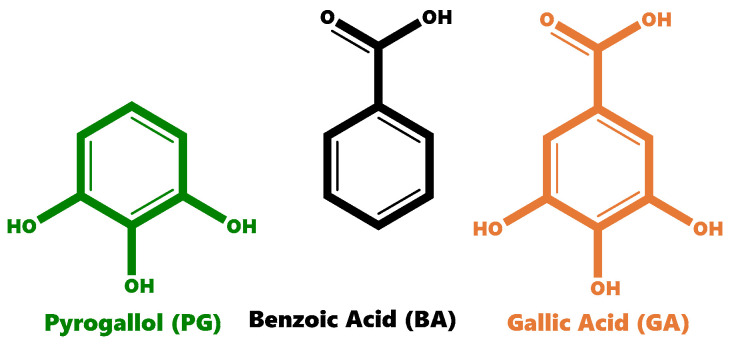
Structure of pyrogallol (**PG**), benzoic acid (**BA**), gallic acid (**GA**).

Carbon quantum dots (**CDs**) are photoluminescent nanoparticles consisting of a carbon-based core covered by surface functionalities [1,2]. Since their discovery in 2004 [3], **CDs** have inspired extensive research due to their remarkable optical properties [4], which make them suitable for a wide range of applications, from biosensing [5] to energy [6,7]. In the literature, **CDs** are broadly classified based on their physicochemical or photophysical properties [8], although these definitions are often used interchangeably and inaccurately. To avoid confusion, most authors have recently adopted the term “**CDs**” to describe all types of carbon-based, quasi-spherical nanomaterials [9]. **CDs** exhibit several advantageous properties including tunable electronic structure, good water dispersion, and photostability [10,11,12]. One of the most significant advantages lies in their ease of preparation via simple top-down or bottom-up approaches, which employ inexpensive, nontoxic, and abundant starting materials. Additionally, their good solubility and easily functionalizable surface, which generally does not require post-synthetic treatments, give them an edge over other carbon-based nanomaterials. Recent studies have explored the surface properties of **CDs** in terms of both non-covalent and covalent interactions with target species [13,14,15,16], which could be exploited in advanced sensing applications, among others. For instance, understanding how **CDs** interact with small molecules commonly used in food preservation could aid the design of sensors based on **CDs** capable of detecting concentrations of these molecules in food and beverages. Such knowledge could also contribute to the development of sustainable intelligent packaging systems that monitor food shelf life. For these reasons, studying the interactions between **CDs** and small molecules currently used, or currently discussed as food additives, such as pyrogallol (**PG**), benzoic acid (**BA**), and gallic acid (**GA**) (see Chart 1) could be of certain interest. These molecules, among other food preservatives, are characterized by similar molecular structures with a benzene ring bearing functionalities that are expected to interact with both surface functionalities and graphitic core of **CDs.**

**CDs** are widely used in sensor designs as cost effective and sustainable alternative to noble metal- and chalcogenide-based nanocrystals [17,18,19,20,21]. On the other hand, **PG**, **BA** and **GA** are used as food preservatives (**FPs**) [22,23,24,25,26]. **BA**, in particular, is one of the most commonly used food preservatives to extend the shelf life of food products [22]. However, in certain aliments like milk, this agent can have harmful effects on human health, such as diarrhea, asthma, convulsions, and abdominal pain [27,28,29,30]. **PG** and **GA** are also recognized for their antioxidant properties [22,23,24,25,26]. **GA**, in particular, is used not only as a natural antioxidant in foods, beverages, and pharmaceuticals but also for its antiviral, antiradical, antibacterial, antimutagenic, anticancer, anti-inflammatory [31], cardiovascular [32], and neuroprotective properties [33,34]. However, upon-long term intake, both **PG** and **GA** can be highly toxic, potentially causing cancer [35,36] and DNA damaging [24,25,26]. It is evident that understanding the interactions between **CDs** and **PG**, **BA**, and **GA**, respectively, is highly relevant for the development of effective **CD**-based sensors. These sensors could detect the presence of these molecules in food products and may be further integrated into sustainable smart packaging to regulate food shelf life.

Thus, based on the aforementioned considerations, in the present work blue-emitting **CDs** were post-functionalized with the promising food preservatives **PG**, **BA**, and **GA** to obtain novel **CD-(FP)** systems. The **CDs** were prepared using a well-established hydrothermal protocol previously developed [37]. UV-vis absorption combined with FTIR analyses of the **CD-(FP)** systems was used to reveal the potential presence of the **FPs** on **CD** surface. Through spectroscopic and electrochemical analyses, we studied the nature of the interactions between **CDs** and the **FP** molecules.

## 2. Materials and Methods

### 2.1. Materials

All used chemicals and solvents were purchased from Sigma (St. Louis, MO, USA), reagent grade and used as received.

### 2.2. Methods

*Synthesis of **CDs***—Nitrogen-doped **CDs** were prepared according to a previously reported route [37]. Citric acid (**CA**) (1 g, 5.2 mmol) and urea (0.95 g, 15.8 mmol) were added to a beaker containing 25 mL of deionized water and stirred until dissolution. The solution was transferred into a Teflon-lined stainless-steel reactor (100 mL) and heated at 200 °C for 24 h. After the reactor was allowed to cool to room temperature, the solution was centrifugated and the supernatant filtrated through a microporous membrane (0.22 μm) to remove the large particles. The reaction water was evaporated in oven, and the solid sample was repeatedly washed with acetonitrile (AcN) followed by centrifugation. TLC analyses were performed to ensure the effective removing of precursor residues and small derivatives. The residual solvent was removed under reduced pressure, and the dark brown solids were stored at 4 °C. The obtained mass is ca 390 mg.

*Synthesis of **CD-(FP)** Systems*—The **CDs** were post-functionalized with the **FP** molecules according to a previously developed method [38]. **CDs** (50 mg) and **FP** molecules (27 μmol) were added to a flask containing 5 mL of methanol (MeOH) and stirred for 24 h at room temperature. The solvent was then evaporated, and the solid was washed three times with sonication in ultrasound bath, employing AcOEt (**BA** and **GA**) and AcN (**PG**) (5 mL each), to remove unreacted **FP** molecules. Then the solid was redissolved in MeOH and the solvent evaporated. The obtained mass was ca 25 mg, that could be stored at 4 °C for months. The **CD-(FP)** systems are soluble in water, and MeOH. The solutions remain stable for months without forming aggregates. The **FP** molecules, **CDs** and **CD-(FP)** systems exhibited good thermal stability up to 50 °C. The reported adsorption rates of the **FP** molecules are the average of three measurements taken at different times to assess reproducibility.

*Characterization Techniques*—UV-vis spectra were collected with a Perkin Elmer (Springfield, IL, USA) Lambda 35 spectrometer; FTIR spectra were recorded using a Nicolet iS10 spectrometer (Thermo Fisher Scientific, Waltham, MA, USA (Life Technologies-IT, n.d.)) equipped with an iTR Smart device (total scan 32, range 4000–800 cm^−1^, resolution 2 cm^−1^). The data were acquired and processed with OMNIC Series Software (Thermo Fisher Scientific, Waltham, MA, USA (Life Technologies-IT)).

For Transmission Electron Microscopy (TEM) imaging, a drop of **CD** suspensions, prepared in MilliQ water, was pipetted on carbon-coated 200 mesh Cu-grids; after 10 min the excess was blotted on filter paper. TEM micrographs were acquired by means of JEOL JEM 2100Plus (JEOL, Tokyo, Japan) operating with an acceleration voltage of 200 kV, and equipped with an 8 megapixel Complemantary Metal-Oxide-Superconductur (CMOS) Rio camera. TEM micrographs were processed with ImageJ Software.

Electrochemical experiments were performed in AcN + 0.1 M tetrabutylammonium perchlorate (TBAP) at 25 °C under nitrogen in three electrode cells. The counter electrode was platinum; unless otherwise stated the reference electrode was a silver/0.1 M silver perchlorate in AcN (Ag/Ag^+^, 0.34 V vs. SCE, −4.73 V vs. vacuum). The working electrode for solution cyclic voltammetry (CV) was glassy carbon (GC) and CVs were performed in 10^−3^ M **FP** molecules solutions. For solid-state CVs GC electrodes 0.2 cm^2^ were used for **CD-(FP)** systems and GC 0.06 cm^2^ for **CDs**. **CD-(FP)** and **CDs** films were casted onto electrodes at 80–90 °C from 2 mg mL^−1^ solutions in MeOH (3 μL). The voltammetric apparatus was Metrohm Autolab 128N potentiostat/galvanostat.

## 3. Results

### 3.1. Characterization of Potential Food Protective Molecules

**Figure 1:**
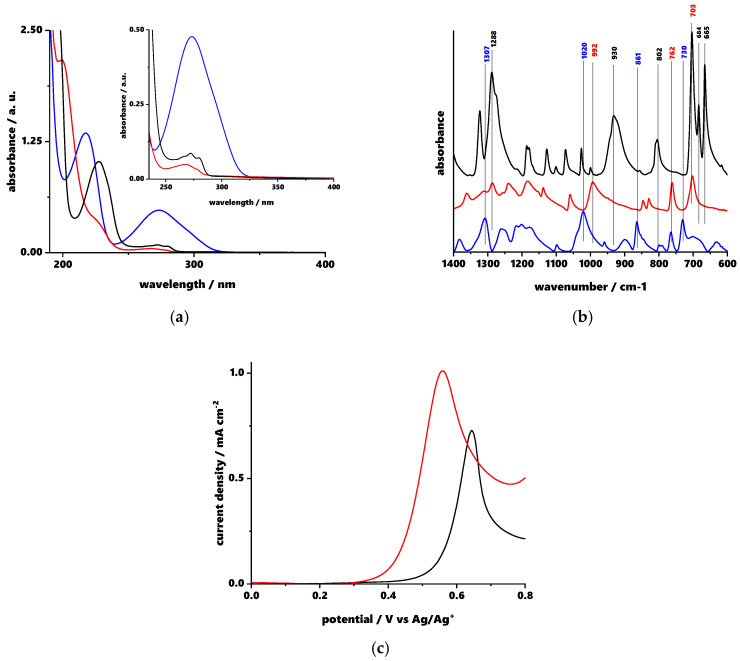
(**a**) UV-vis absorption spectra of **PG** (red line), **BA** (black line), and **GA** (blue line) solutions 5 × 10^−4^ M in MeOH. Inset: Magnified UV-vis absorption B-band of **PG** (red line), **BA** (black line), and **GA** (blue line); (**b**) FTIR spectra of **PG** (red line), **BA** (black line), and **GA** (blue line); (**c**) Sigle-sweep Cyclic Voltammograms of **PG** (red line), and **GA** (black line) solutions 10^−3^ M in AcN + TBAP on GC electrode. Scan rate 0.1 v s^−1^.

*UV-vis analyses*—The UV–visible absorption spectrum of **PG** (Figure 1a and Table 1) exhibits two bands at 200 nm and 267 nm, accompanied by two shoulders at 223 nm and 277 nm, respectively. The bands originate from π-π* transitions in the benzene ring. According to Braude’s designation, they are the E and B bands, respectively [39]. Compared to benzene, both bands shift to longer wavelengths due to the auxochromic OH groups substitution on the ring, and the two shoulders result from the loss of the fine structure of benzene caused by n-π conjugation induced by the OH substituents [39]. On the other hand, the direct attachment of the unsaturated chromophore carboxylic group to the benzene ring in **BA** produces the appearance of the K-band at 228 nm in its UV-vis response (Figure 1a, Table 1) [39] and a bathochromic shift of the B-band at 272 nm compared to **PG**. The B-band exhibits a fine structure, likely due to the absence of auxochromic OH groups substitution. In **GA**, the functional groups of **PG** and **BA** appear on the same ring and synergistically influence the absorption response of the molecule (Figure 1a and Table 1). The K-band shifts to 217 nm compared to **BA**, likely due to the overlap with the E-bands, which are bathochromically displaced by auxochromic OH group substitution [35]. The B-band at 272 nm increases in intensity and loses its fine structure. The extinction coefficients of the three molecules in MeOH solutions were estimated at their B-bands (Table 1). The ε value of **GA** is nearly an order of magnitude higher than those of **PG** and **BA**, likely due to the extended conjugation caused by the presence of both the carboxylic and OH groups.

*FTIR analyses*—Figure S1 shows the full FTIR spectra of all three **FP** molecules. In this section, the spectral range from 1400 cm^−1^ to 600 cm^−1^ is discussed in detail (Figure 1b) because is unaffected by the distinctive bands of **CDs**, as will be demonstrated below. The FTIR response of **PG** in this range (Figure 1b) shows the characteristic bands of the phenolic OH-groups, including the C-O stretching band at 992 cm^−1^ coupled with the adjacent C-C stretching band at 1056 cm^−1^ [39,40]. The spectrum also shows the characteristic bands for the benzene ring: the C-H out-of-plane bending bands at 846, 827, 762, and 703 cm^−1^ [35,36]. In contrast, the **BA** spectrum (Figure 1b) shows the C-O stretching band of the carboxylic group at 1288 cm^−1^ and the benzene ring’s characteristic C-H out-of-plane bending bands at 802, 703, and 665 cm^−1^. The characteristic band of dimeric carboxylic acid is also present, which is the out-of-plane bending of the bonded O-H at 930 cm^−1^ [39]. In **GA**, the presence of the functional groups of both **PG** and **BA** influences its FTIR response (Figure 1b). In particular, the C-O stretching bands of the carboxylic group and phenol are shifted to higher wavenumbers compared to **PG** and **BA**: 1307 cm^−1^ for the carboxylic group and 1020 cm^−1^ for phenol. The C-H out-of-plane bending bands of the benzene ring are at 762 and 730 cm^−1^, and the out-of-plane bending band of the bonded O-H in dimeric carboxylic acids is at 861 cm^−1^.

*Electrochemistry*—The cyclic voltammograms of **PG** in AcN and TBAP (see Figure 1c and Figure S2a) exhibited one reversible oxidation process at 0.56 V vs. Ag/Ag^+^ (see Table 1). The peak width of 47 mV indicates a fast electron transfer rate on the GC electrode. In **GA**, the presence of the electron-attractive carboxylic group influences the electrochemical response (see Figure 1c and Figure S2b). Specifically, its oxidation process is 80 mV more positive than that of **PG** and has a peak width of 31 mV (Table 1). These electrochemical processes are due to the two-electron oxidation of the OH groups to their corresponding quinone forms [26,41]. Neither molecule exhibits reduction processes within the electrochemical window of the GC electrode in AcN. In contrast, **BA** shows no oxidation or reduction response in the GC electrode’s electrochemical window in AcN.

### 3.2. CDs

**Figure 2:**
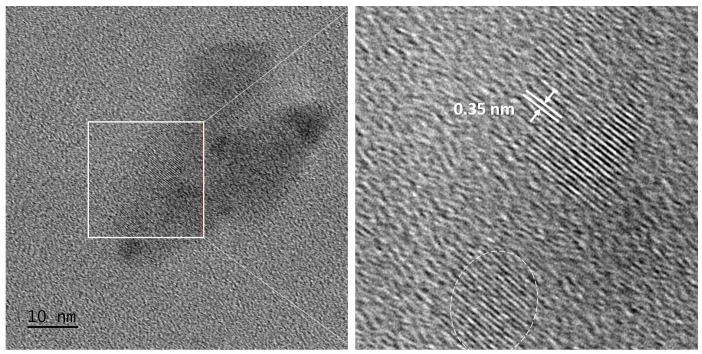
HRTEM micrographs of **CDs**: The resolved lattice fringes correspond to (002) graphitic planes.

Blue-emitting **CDs** were prepared through a hydrothermal strategy previously developed [33]. High-resolution transmission electron microscopy (HRTEM) revealed that the **CDs** consist of spherical nanoparticles ranging from 5 to 13 nm in diameter (Figure S3), with an average diameter of 8 nm (Figure 2) [39]. The planar lattice fringes exhibit interplanar spacing of 0.35 nm, which is highlighted in white in Figure 2 and corresponds to the (002) plane of the graphitic crystal structure [42]. As reported in our previous works [15,37,43] the FTIR response of **CDs** (Figure S4a) indicates that the functional groups at the edges of their graphitic basal planes are primarily amides and ammonium carboxylates. These groups have distinctive vibrational bands ranging from 3500 to 1350 cm^−1^, which limits potential interference with the FTIR responses of the **FP** molecules reported in the previous section. Additionally, the reported [37] **CD** UV-vis absorption response (Figure S4b) exhibits a shoulder at 230 nm, which is attributed to the π-π* transition of the graphitic core, as well as a peak at 320 nm, which is attributed to the n-π* transitions of the C=O or C=N functionalities. Regarding possible interference with the aforementioned **FP** molecules, it should be noted that the n-π* transition band of the **CDs** is 53 and 48 nm lower in wavelength than the B-bands of **PG** and **BA** and **GA**, respectively. The reported photoluminescence emission of **CDs**, upon excitation at the n-π* transition, peaks at 420 nm [37]. This indicates deep blue emission with a quantum yield of 21.5%. Finally, we observed that **CDs** do not present electrochemical responses in the potential range of interest of **PG** and **GA** [37].

### 3.3. CD-(FP) Systems

**Scheme 1:**
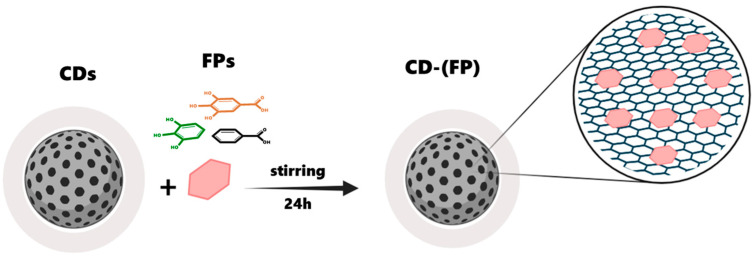
Scheme of the formation of CD-(FP) systems.

Scheme 1 shows the post-functionalization of **CDs** with the **FP** molecules. The **CD-(FP)** systems are prepared according to a previously developed method [38], detailed again in the Experimental Section. The reciprocal amounts of **CDs** and **FP** molecules were determined according to a study reported by Guldi, Prato et al. [15] on the post-functionalization of **CDs**. Excess quantities of **FP** molecules are removed by washing the **CD-(FP)** systems with solvent. As discussed in Section 3.1, the FTIR responses of the **CD-(FP)** systems were analyzed in the spectral range from 1400 cm^−1^ to 600 cm^−1^.

**Figure 3:**
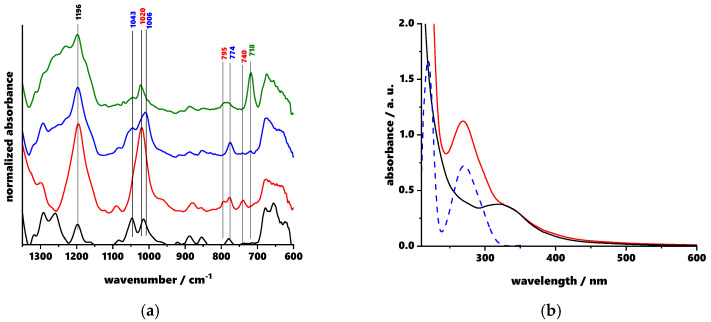
(**a**) FTIR spectra of bare **CDs** (black-line), and **CD-(PG)** (blue line), **CD-(BA)** (green line), and **CD-(GA)** (red line) systems; (**b**) UV-vis spectra of **CD-(GA)** (red line) and **CD** (black line) solutions of 1 mg mL^−1^ in MeOH. **GA** component (dashed blue line) results from the difference between **CD-(GA)** and **CD** spectra.

***CD-(BA)** systems*—The UV-vis response of the **CD-(BA)** systems (Figure S5a) shows the characteristic n-π* transition band of CDs at approximately 320 nm, with a shoulder at approximately 275 nm. Subtraction of the UV-vis response of the bare CDs reveals the appearance of the characteristic B and K bands of **BA** at 228 and 272 nm, respectively. The estimated percentage of the adsorbed **BA** is approximately 4% (*w*/*w*) (ca 300 μmol/g). The FTIR response (Figure 3a) shows the typical C-H out-of-plane bending bands of the benzene ring at 718 and 786 cm^−1^, as well as a new band at 1196 cm^−1^. Absent are the C-O stretching bands of the carboxylic group and the out-of-plane bending band of the bonded O-H in dimeric carboxylic acids.

***CD-(PG)** systems*—Similarly to the aforementioned **CD-(BA)** system, the absorption response of the **CD-(PG)** systems (Figure S5b) reveals the appearance of the typical B band of **PG** at approximately 268 nm and the shoulder of its E band at approximately 223 nm upon the subtraction of the UV-vis response of the bare **CDs**. The estimated percentage of the adsorbed **PG** is again approximately 4% (*w*/*w*) (ca 300 μmol/g). The FTIR response (Figure 3a) shows the characteristic C-O stretching bands of the phenol OH groups at 1006 cm^−1^ coupled with the C-C stretching band at 1043 cm^−1^, as well as the C-H out-of-plane bending bands of the benzene ring at 774 cm^−1^ and a new band at 1196 cm^−1^. In contrast to the free **PG**, the **CD-(PG)** systems do not exhibit electrochemical responses (Figure S5a).

***CD-(GA)** systems*—Similarly to **CD-(BA)** and **CD-(PG)**, the UV-vis response of **CD-(GA)** (Figure 3b) exhibits the K and B bands of **GA**, appearing at approximately 270 and 218 nm, respectively, when the response of the bare **CDs** is subtracted. The estimated percentage of adsorbed **GA** is approximately 6% (*w*/*w*) (ca 2000 μmol/g). The FTIR response (Figure 3a) shows the C-O stretching bands characteristic of phenolic OH-groups at 1020 cm^−1^, as well as the out-of-plane C-H bending bands of the benzene ring at 740, 774, and 795 cm^−1^ and the new band at 1196 cm^−1^, identical to that of **CD-(PG)** and **CD-(BA)**. Absent are the C-O stretching bands of the carboxylic group and the out-of-plane bending band of the bonded O-H in dimeric carboxylic acids, similar to **CD-(BA)**. Like **CD-(PG)**, **CD-(GA)** systems do not exhibit electrochemical responses.

## 4. Discussion

The aforementioned results on **CD-(FP)** systems allow for remarks concerning the nature of **CD**/**FP** interactions. UV-vis spectroscopy shows the presence of the **FP** molecules on the **CD** surface. All **CD-(FP)** spectra exhibit the characteristic absorption bands of the respective **FP** molecules (see Figure S5a,b and Figure 3b), and the estimated degree of coverage was found to be similar for all the three **FP** molecules (4–6% (*w*/*w*) approximately 2000–300 μmol/g). These values are comparable to those reported by Guldi, Prato et al. [11] in their work on the post-functionalization of **CDs**. FTIR analyses largely support the UV-vis data, exhibiting the bands typical of the various **FP** molecules in the tested systems, i.e., **PG**, **BA**, and **GA**. However, the typical out-of-plane C-H bending bands of the benzene ring (Figure 3a) are shifted to a higher wavenumber, compared to those of the free molecules. Furthermore, the spectra of **CD-(PG)** and **CD-(GA),** also clearly show the C-O stretching bands of the phenolic OH groups (Figure 3a). Notably, while the wavenumber values for these latter bands of **CD-(GA)** remain unchanged compared to free **GA**, those of **CD-(PG)** shift to higher wavenumbers. With respect to the bands indicating the C-O stretching of the carboxylic group and the out-of-plane bending of the bonded O-H in dimeric carboxylic acids for free **BA** and **GA**, the spectra of **CD-(BA)** and **CD-(GA)** no longer show those bands. Conversely, the spectra of all **CD-(FP)** systems exhibit a new band at 1196 cm^−1^ (Figure 3a). To the best of our knowledge, this band is a characteristic of the systems under study. We performed thermal stability tests on the **FP** molecules, **CDs**, and **CD-(FP)** systems to rule out the possibility of sample degradation causing the band. All examined samples remained stable up to 50 °C.

**Scheme 2:**
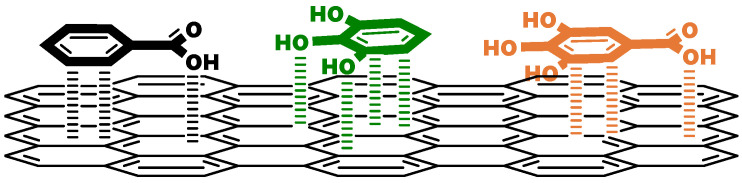
Electronic interactions between **FP** molecules and **CDs**.

The new band falls within the spectral range of both the C-H in-plane bending of the benzene ring and the C-O stretching of the OH groups [39,40]. It is more likely attributable to the former than the latter because BA does not display a phenolic OH group (see Chart 1). The presence of this new band, along with the shift observed in the C-H out-of-plane bending bands of the benzene ring, allows for hypothesizing that the interaction between the **FP** molecules and the **CDs** is non-covalent. Spectral data suggest that it occurs through electronic interaction between the delocalized electronic systems of the benzene rings of the **FP** molecules and the graphitic structure of the **CDs** exposed at the surface (see Scheme 2). Such interaction would be in line with the C-H in-plane bending vibrations of the ring appearing to be favored over the out-of-plane vibrations. Additionally, the fact that the band at 1196 cm^−1^ is present in all **CD-(FP)** systems independently of their different functional groups supports the presence of electronic interactions rather than covalent or ionic bonding. The latter is also less likely given the functional groups available on the surface of the **CDs**. All the **FP** molecules have an acidic character, so it could be expected that the surface carboxylates would make attachment more difficult. Yet, electronic interaction seems favorable and strong enough to lead to the observed complex systems (Scheme 2). This hypothesis aligns with the findings of Güner et al. [44], who reported that non-covalent electronic interactions between conjugated molecular systems and carbon nanotubes are favored over covalent or electrostatic interactions. Additionally, Arcudi et al. [9] report on electronic non-covalent interactions between conjugated molecular systems and the carbonaceous core of **CDs** in their review work on supramolecular chemistry of **CDs**.

Furthermore, the absence of the typical vibrational bands of the carboxylic group in the **CD-(BA)** and **CD-(GA)** spectra suggests an additional interaction of the carboxylic group.

In these two systems, this could be understood assuming that they may undergo hydrogen bonding with the nitrogen atoms (**N-atoms**) present in the honeycomb matrix of **CDs** in the form of pyridinic, pyrrolic, and graphitic N atoms [14,33,39]. Conversely, in **CD-(PG)** systems, the phenolic OH groups appear to interact with the **CDs** in an additional way, as we observed a shift in their bands. This interaction appears to be absent in **CD-(GA)** because the OH bands do not shift, with respect to pure **GA**; the interaction with the carboxylic group seems to be dominant. The electrochemical responses of **CD-(PG)** and **CD-(GA)** support the FTIR analyses. The CV response of both systems does not show the oxidation processes observed in free **PG** and **GA** (Figure S6a and Figure 1c). Therefore, we can infer that the interaction between the **PG** and **GA** benzene rings and the **CDs** prevents their oxidation to the corresponding quinones. This hypothesis is also confirmed by the CV responses of free **PG** and **GA** on GC electrodes modified with a cast film of **CDs** (Figure S6b). Their oxidation peak is suppressed (Figure S6b). We excluded the effect of electrode passivation induced by **CDs** on the GC electrode because we observed the electrochemical responses of other conjugated systems, such as fluorenes, on **CD**-modified GC electrodes. Therefore, we attribute the observed phenomenon to possible electron transfer suppression due to interactions between the molecules and the **CDs**. It seems that the aforementioned electronic interactions hinder the formation and stabilization of the **PG** and **GA** quinone forms reported to form during oxidation [26,36] by blocking their benzene rings on the surface of **CDs**.

## 5. Conclusions

In this study, we explored the interaction between **CDs** and the promising **FP** molecules pyrogallol (**PG**), benzoic acid (**BA**), and gallic acid (**GA**). We also examined the nature of these interactions. UV-Vis and FTIR analyses of the **CD-FP** systems revealed the presence of the molecules on the **CD** surface. The estimated degree of adsorption is approximately 4% (*w*/*w*) (ca 300 μmol/g) for the **CD-(PG)** and **CD-(BA)** systems and 6% (*w*/*w*) (ca 2000 μmol/g) for the **CD-(GA)** system. These values are consistent with those reported in the literature [11]. The presence of a new FTIR band at 1196 cm^−1^, common to all **CD-(FP)** systems (Figure 3a), suggests that adsorption of the **FP** molecules onto the **CD** surface occurs via electronic interactions of delocalized electron systems through the graphitic honeycomb structure rather than through formation of ion pairs or covalent bonds with the CD surface’s functional groups (see Scheme 2). In the **CD-(BA)** and **CD-(GA)** systems, these electronic interactions are further strengthened by hydrogen bonding involving the carboxylate groups in **BA** and **GA**. In the **CD-(PG)** systems, they are strengthened by the phenolic OH groups. Additionally, electrochemical determinations show that the intense interactions between **CDs** and **FP** molecules influence the oxidation of **PG** and **GA** to their corresponding quinone forms.

In light of recent advances in the dynamics of two-dimensional nanomaterials [45,46,47], these results are expected to be useful for developing sensing devices based on CDs. This knowledge can be used to design sensing devices using different strategies, ranging from food analysis to developing smart, sustainable food packaging. Further studies are on course to investigate the interactions between CDs and biocompatible molecules.

## Figures and Tables

**Table 1:** UV-vis absorption maxima (λ_max_), molar extintion coefficient (ε) in MeOH, oxidation potential (E_ox_) and peak width (ΔE) of **PG**, **BA**, and **GA**.

Sample	λ_max_ (nm)	ε (M^−1^ cm^−1^)	E_ox_ (V)	ΔE (mv)
**PG**	200, 223(s); 267, 277(s)	1 × 10^3^	0.56	47
**BA**	228; 272	1.8 × 10^3^	-	-
**GA**	217; 272	9.6 × 10^3^	0.64	31

## Data Availability

Data are contained within the article and Supplementary Materials.

## Supplementary Materials

The following supporting information can be downloaded at: https://www.mdpi.com/article/10.3390/nano15181457/s1, Figure S1: FTIR spectra of the **PG**, **BA**, **GA**; Figure S2: Cyclic Voltammograms of **PG** and **GA**; Figure S3: Size distribution of **CDs**; Figure S4: FTIR and UV-vis spectra of **CDs**; Figure S5: UV-vis spectra of **CD-(PY)** and **CD-(BA)**; Figure S6: Cyclic Voltammograms of **CD-(PG)** systems cast films and **PG** on GC electrode modified with **CDs** cast film.
