# Supplementary material for: Carbon Quantum Dots Interactions with Pyrogallol, Benzoic Acid, and Gallic Acid: A Study on Their Non-Covalent Nature
Source: Nanomaterials (Basel). 2025 Sep 22;15(18):1457. doi: 10.3390/nano15181457 (PMC12472236; doi:10.3390/nano15181457)
Supplement: Supplementary file 1 [file nanomaterials-15-01457-s001.zip › nanomaterials-3841080-supplementary.pdf]

# Carbon Quantum Dots Interactions with Pyrogallol, Benzoic Acid, and Gallic Acid: A Study on their Non-Covalent Nature

Laura Andria[1], Giancarlo Capitani[2], Barbara La Ferla[1,2], Heiko Lange[2,3,4], Melissa Saibene[5], Luca Zoia[2] and Barbara Vercelli[1]*

[1]*Istituto di Chimica della Materia Condensata e di Tecnologie per l'Energia, CNR-ICMATE, Via Cozzi, 53 I–20125 Milano (Italy)*

[2]*Dipartimento di Scienze dell'Ambiente e della Terra, Università degli Studi di Milano-Bicocca, Piazza della Scienza, 1 I–20126 Milano (Italy)*

[3]*Biochemical Process Engineering, Division of Chemical Engineering, Department of Civil, Environmental and Natural Resources Engineering, Luleå University Technology, SE-97187 Luleå (Sweden)*

[4]*NBFC- National Biodiversity Future Center I-90133 Palermo (Italy)*

[5]Piattaforma di Microscopia, Università degli Studi di Milano-Bicocca, Piazza della Scienza 3, I-20126 Milano (Italy)

**General Contents**

**FTIR and electrochemical data of BA, Py and GA** **page S2**

**FTIR and UV-vis and TEM data of CDs** **page S3**

**UV-vis data of Cds-(BA) and CDs-(Py) and electrochemical data Of CDs-(Py) and Py** **page S4**

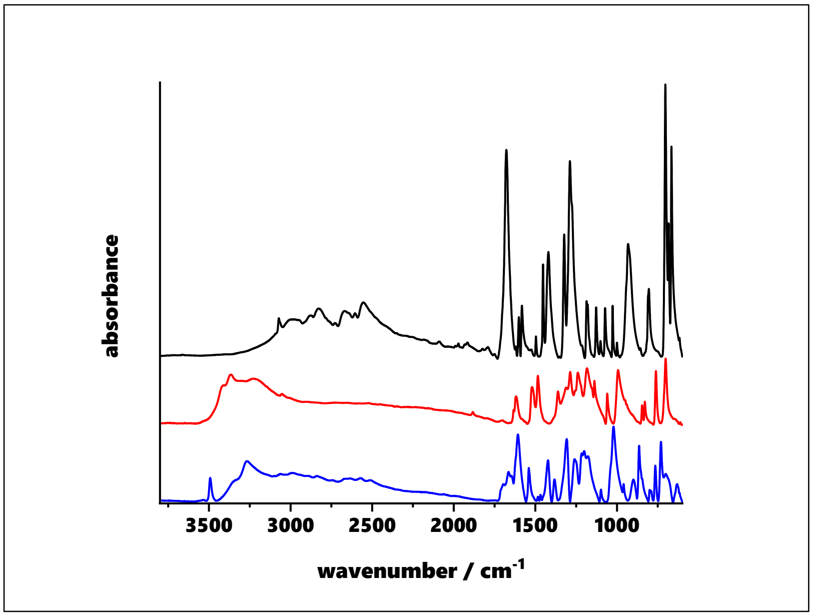


**Figure S1** FTIR spectra of the **PG** (red line), **BA** (black line), and **GA** (blue line).

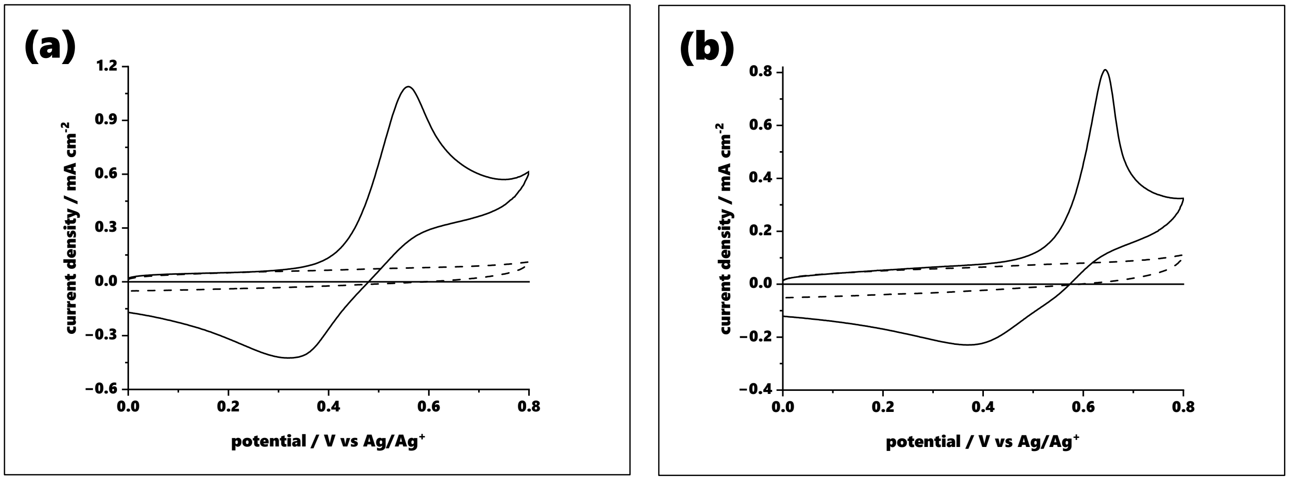


**Figure S2.** Cyclic Voltammograms of (a) **PG** and (b) **GA** solutions $10^{-3}$ M in ACN + TBAP on GC electrode; v = 0.1 V $s^{-1}$. Dashed line GC background signal.

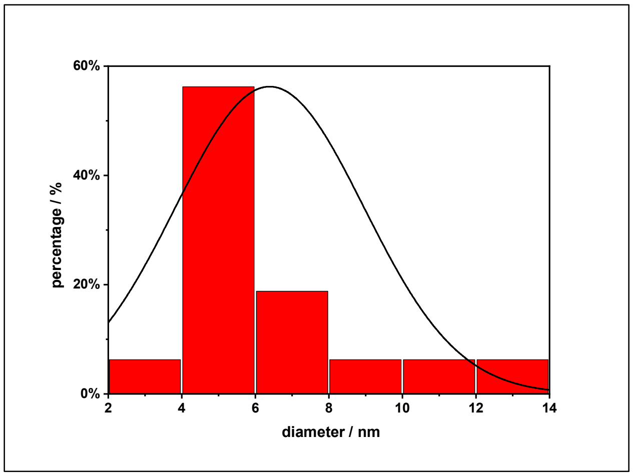


**Figure S3.** Size distribution of CDs

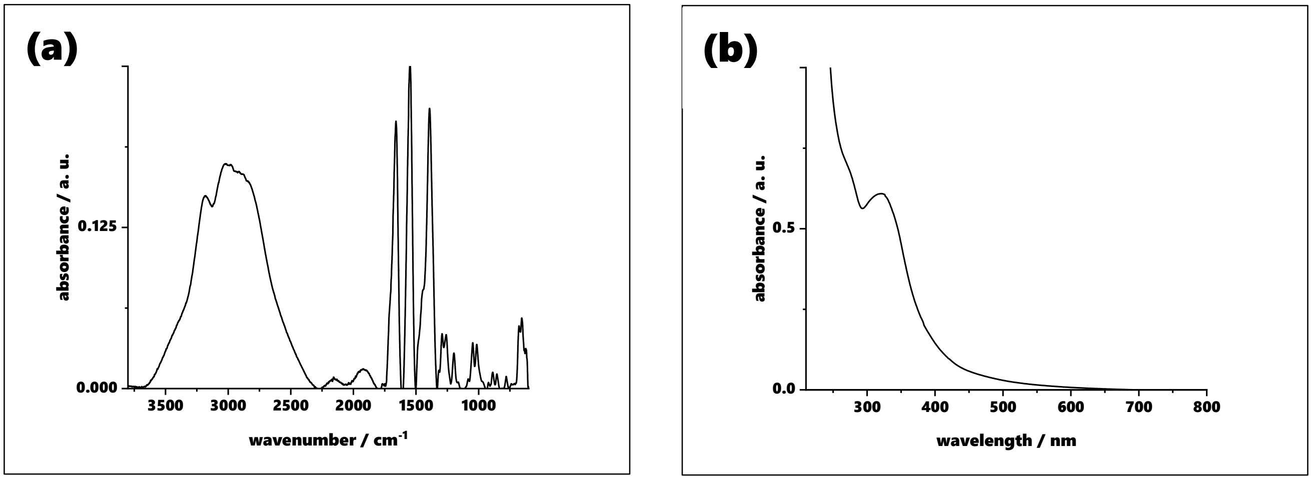


**Figure S4.** (a) FTIR spectrum of **CDs**; (b) UV-vis absorption spectrum of **CDs** solution 1 mg $mL^{-1}$ in MeOH;

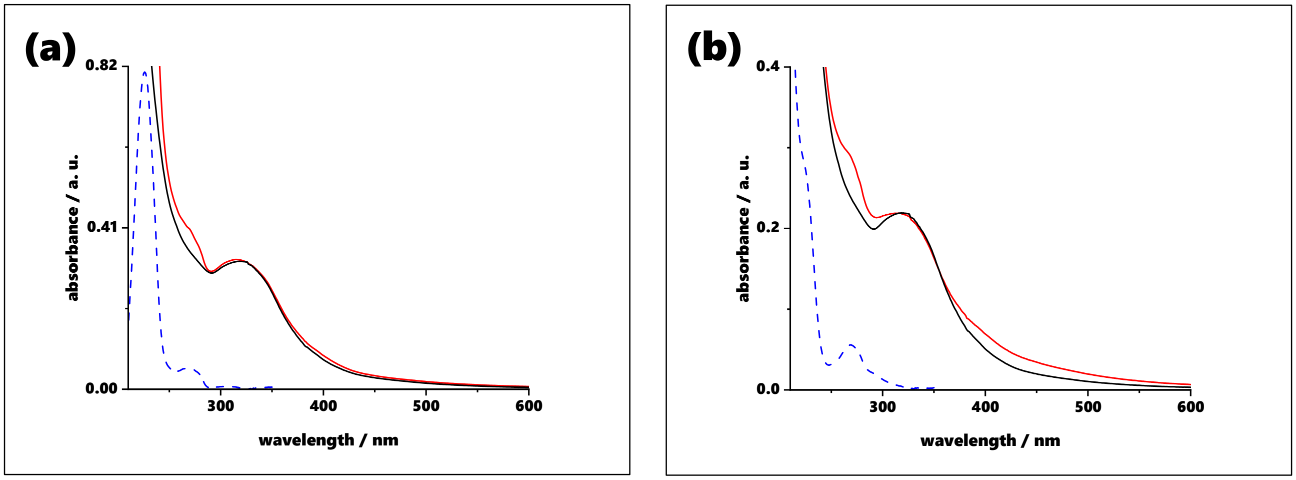


**Figure S5.** (a) UV-vis spectra of **CD-(BA)** (red line) and **CDs** (black line) solutions 1 mg mL$^{-1}$ in MeOHand. **BA** component (dashed blue line) result of the difference between **CD-(GA)** and **CDs** spectra.; (b) UV-vis spectra of **CD-(PG)** (red line) and **CDs** (black line) solutions 1 mg mL$^{-1}$ in MeOH. **PG** component (dashed blue line) result of the difference between **CD-(GA)** and **CDs** spectra.

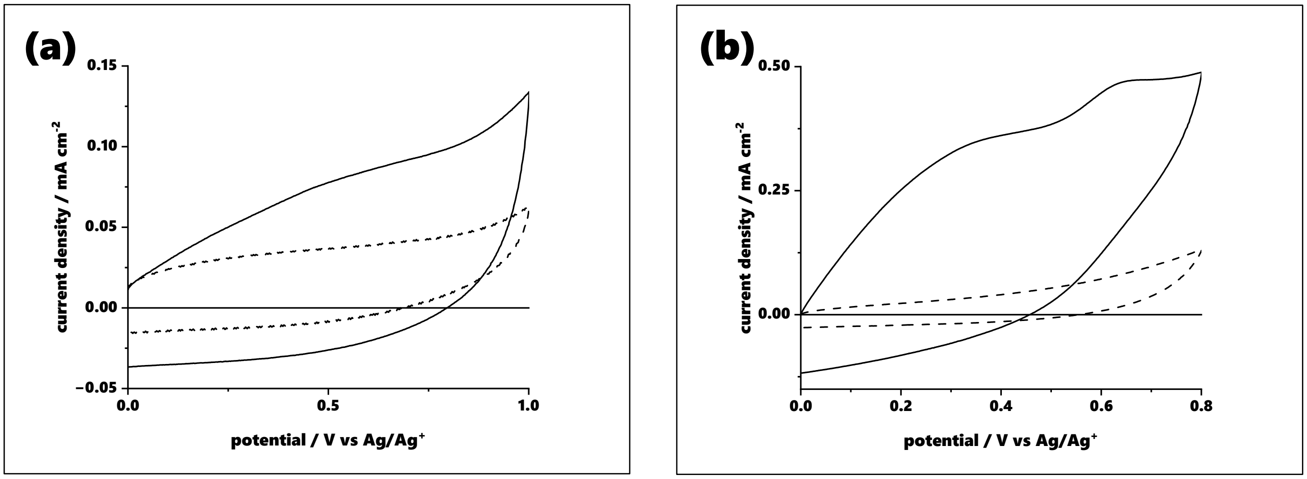


**Figure S6.** (a) Cyclic Voltammogram of **CD-(PG)** cast film on GC electrode (0.2 cm$^{2}$) in AcN + TBAP; v = 0.1 V s$^{-1}$. Dashed line GC background signal; (b) Cyclic Voltammogram of **PG** solution 10$^{-3}$ M in ACN + TBAP on GC electrode (0.06 cm$^{2}$) modified with **CD** cast film. v = 0.1 V s$^{-1}$. Dashed line GC modified with **CD** cast film background signal.
